# Mitochondrial Dynamics in the Brain Are Associated With Feeding, Glucose Homeostasis, and Whole-Body Metabolism

**DOI:** 10.3389/fendo.2020.580879

**Published:** 2020-11-09

**Authors:** Jessica L. Haigh, Lauryn E. New, Beatrice M. Filippi

**Affiliations:** Faculty of Biological Sciences, School of Biomedical Sciences, University of Leeds, Leeds, United Kingdom

**Keywords:** ****mitochondrial dynamics, brain, feeding, glucose—insulin, metabolism

## Abstract

The brain is responsible for maintaining whole-body energy homeostasis by changing energy input and availability. The hypothalamus and dorsal vagal complex (DVC) are the primary sites of metabolic control, able to sense both hormones and nutrients and adapt metabolism accordingly. The mitochondria respond to the level of nutrient availability by fusion or fission to maintain energy homeostasis; however, these processes can be disrupted by metabolic diseases including obesity and type II diabetes (T2D). Mitochondrial dynamics are crucial in the development and maintenance of obesity and T2D, playing a role in the control of glucose homeostasis and whole-body metabolism across neurons and glia in the hypothalamus and DVC.

## Introduction

Mitochondria are essential for the control of energy metabolism and their dysfunction plays a key role in the pathophysiology of obesity and type II diabetes (T2D). They are dynamic organelles that change morphology in response to energy demand and supply *via* fission and fusion. Fission is mediated by the GTPase dynamin-related protein 1 (DRP1) and occurs when mitochondria are damaged or subjected to high levels of cellular stress (1, 2). DRP1 is recruited to the outer mitochondrial membrane (OMM) where it oligomerizes to form a ring structure to constrict the mitochondrion and fragment it (3, 4); this allows for a reduction in ATP content when energy supply exceeds demand. Fusion is also mediated by GTPases which include mitofusin 1 (MFN1), mitofusin 2 (MFN2), and optic atrophy protein 1 (OPA1) (5–7). MFN1 and MFN2 are anchored to the OMM and mediate fusion there, while OPA1 is involved in inner mitochondrial membrane (IMM) fusion. Mitochondrial fusion serves to repair damaged mitochondria by fusing them with a functional one and also increases ATP production to meet metabolic demand (2). Mice lacking *Drp1*, *Mfn1*, *Mfn2*, or *Opa1* are embryonic lethal (5, 8–10), demonstrating their essential role in development and highlighting their potential to influence metabolism if expression levels change.

Circuits within the central nervous system (CNS) control whole-body energy metabolism. The hypothalamus and the dorsal vagal complex (DVC) have been highlighted as crucial regulatory centers of metabolism, and dysfunction of mitochondria in these regions has been implicated in metabolic disorders. The arcuate nucleus (ARC) of the hypothalamus and area postrema (AP) of the DVC are sites with increased blood–brain barrier (BBB) permeability allowing for detection of circulating nutrients, such as glucose and free fatty acids, and hormones that have restricted entry to other brain regions (11). The ability to sense humoral signals means these regions can respond to environmental cues and adapt metabolism accordingly. Within the ARC, anorexigenic proopiomelanocortin (POMC)- and orexigenic agouti-related peptide (AgRP)-expressing neurons regulate energy expenditure, glucose homeostasis, and food intake in an antagonistic fashion. Glial cells are also involved in body weight homeostasis, glucose metabolism, and obesity, with roles for both microglia and astrocytes (12–16).

In this review, we discuss the contribution of mitochondrial dynamics and dysfunction in the brain to the central control of metabolism and glucose homeostasis and their role in the metabolic disorders obesity and T2D.

## Brain Inflammation, ER Stress and Mitochondria in Obesity and T2D

The body must respond and adapt to changing levels of energy input and availability by altering metabolism. This occurs at the molecular and transcriptional levels to give systems level changes and alterations in behavior. Mitochondria respond to nutrient availability by fusion or fission to maintain homeostasis; however, these processes can be disrupted by metabolic disease. Obesity and its many co-morbidities are associated with chronic inflammation, ER stress, and disrupted mitochondrial dynamics in several tissues [reviewed in (17–20)].

Obesity triggers a chronic low-grade inflammatory response in multiple organs which presents a complex challenge for deciphering the disease mechanisms. The mitochondria produce adenosine triphosphate (ATP) using nutrients and oxygen in a series of stepwise reactions that occur at the inner mitochondrial membrane (IMM). This process is the main source of reactive oxygen species (ROS), important for cell functions and acting as a signaling molecule; however, high ROS levels are associated with cell damage. Excessive nutrient intake overloads the mitochondria with glucose and fatty acids leading to an increase in ROS production and associated oxidative stress in the skeletal muscle (21, 22). Obesity leads to reduced *β*-oxidation, a process where fatty acids are broken down to generate acetyl-CoA for use in the citric acid cycle (23). The reduction in *β*-oxidation impairs cellular functions *via* increased triacylglycerol synthesis and ectopic deposition of lipids; it also causes oxidative stress *via* an increase in lipid peroxidation byproducts, increased nitric oxide synthase, and excess ROS (24). High-fat diet (HFD) in rats reduced the activity of antioxidant enzymes superoxide dismutase, glutathione peroxidase, and catalase in the brain, thus further increasing ROS levels (25). When ROS production is exacerbated, for example by increased lipid availability, mitochondrial dysfunction is induced, and ATP production is decreased (26). ROS activate several transcription factors including NF-*κ*B, which is the primary mediator of the inflammatory response. HFD in mice increased NF-*κ*B activity in multiple tissues (27) and activated inflammatory pathways in the hypothalamus leading to insulin resistance (28, 29). Levels of macrophages and T cells are increased in the skeletal muscle of obese patients with insulin resistance or T2D (30, 31), and short-term overfeeding in healthy subjects increased macrophage markers in the skeletal muscle and impaired insulin signaling (32), illustrating that inflammatory processes are also triggered by metabolic disorder in humans. Another family of inflammatory molecules c-Jun N-terminal kinases (JNKs) are activated in obesity; when JNK1 KO mice were fed HFD they gained less weight, had smaller adipocytes, and improved insulin sensitivity compared to wildtypes (33). Immune responses throughout the body and metabolic regulation are highly integrated, and the proper function of each is dependent on the other.

The endoplasmic reticulum (ER), a complex protein folding and trafficking organelle, interacts with the mitochondria and is another source of ROS. The ER can transduce signals from lipid metabolites and cytokines to stress kinases and plays an important role in insulin resistance (34). ER-mediated ROS production is increased in insulin resistant non-diabetic obese patients and T2D patients (35, 36). During cellular stress, as seen in T2D patients, ER function is disrupted by the unfolded protein response (UPR); this leads to calcium leakage from the ER which interferes with electron transfer in the electron transport chain and ultimately induces mitochondrial ROS production (37). There are three major transducers of the UPR: protein kinase RNA (PKR)-like ER kinase (PERK), inositol-requiring enzyme 1 (IRE1), and activating transcription factor 6 (ATF6). Downstream signaling leads to transcription of UPR target genes including upregulation of molecular chaperones and folding catalysts to increase the folding capacity of the ER, conveying a protective effect for cell survival. The UPR is an acute response to re-establish cellular homeostasis following stress; however, if sustained chronically it can lead to disease including obesity, T2D, and neurodegenerative disorders to name a few (38). ER stress is triggered in obese subjects by the release non-esterified fatty acids from visceral adipose tissue which causes the increased expression of cytokines and activation of PERK (39). Phosphorylated PERK modulates insulin responsiveness in adipose tissue through generation of phosphatidic acid, an inhibitor of insulin action (40). Furthermore, PERK-dependent mechanisms can induce positive ER stress feedback that perpetuates the process, contributing to the development of insulin resistance (39). A recent *in vitro* study found that ER stress and the UPR cause insulin resistance by depleting the cell surface population of the insulin receptor, impairing the transport of newly synthesized proreceptors (41). This effect of ER stress on trafficking insulin receptors to the surface may account for the decrease in insulin receptors seen in T2D patients. ER stress was detected in the hypothalamus of leptin-deficient neonate mice (*ob/ob*) before the development of obesity at postnatal day 10 (P10) (42). Administration of the ER stress-relieving compound TUDCA during a critical developmental window for the hypothalamus (P4–P16) provided beneficial effects on body weight, food intake, adiposity, and glucose homeostasis that persisted into adulthood (42). ER stress throughout life therefore has a strong impact on metabolic regulation, including the hypothalamic circuits involved in feeding behavior.

Along with an upregulation in mitochondrial ROS production, dysfunction in mitochondrial dynamics is also associated with metabolic disease. A decrease in number of mitochondria and reduction in bioenergetic capacity have been measured in T2D patients (43). Reduced expression of the mitochondrial fusion mediator MFN2 was seen in skeletal muscle of obese Zucker rats and non-obese T2D patients (44, 45). Both genetically obese and diet-induced obese mice were found to have smaller and shorter mitochondria in the skeletal muscle, while inhibition of fission improved systemic insulin sensitivity (46). Rats on HFD for 8 weeks presented with impaired mitochondrial respiration and insulin resistance in hepatocytes (47). Treatment of hepatocytes with high glucose caused the mitochondria to become smaller and shorter; when a dominant negative form of DRP1, Drp1-K38A (a point mutation at Lysine-38 to Alanine in the catalytic site) was expressed in the cells, mitochondrial fission was prevented following glucose administration (48). In the adipose tissue of morbidly obese patients, an inverse relationship was found between the regulation of inflammation and mitochondrial function (49), and adipocytes in obese and diabetic mouse models had decreased mitochondrial mass and function (50).

Clear roles for aberrant mitochondrial dynamics have therefore been shown in peripheral tissues during the development of insulin resistance and obesity. Oxidative stress and ER stress are associated processes that both result in inflammation and are linked to the development of insulin resistance in many tissues including liver, skeletal muscle, adipose tissue, and brain. Mitochondria are crucial in maintaining energy balance and are therefore key players in the pathophysiology of metabolic disorders in peripheral organs as well as in the central nervous system.

## Energy Homeostasis and Mitochondrial Dynamics in the Hypothalamus

The mediobasal hypothalamus (MBH) is critical for the regulation of appetite, feeding behavior, and body weight. Specific populations of neurons within the ARC respond to circulating hormones including insulin, leptin, and ghrelin to maintain energy homeostasis (51). AgRP/NPY neurons release GABA and directly inhibit POMC neurons (52). The binding of insulin in the ARC causes hyperpolarization of AgRP neurons *via* PI3K/Akt pathway and subsequent ATP-sensitive K^+^ (K_ATP_) channel activation, alleviating the inhibitory constraints on POMC neurons (53, 54). Insulin binding in the ARC causes a decrease in hepatic glucose production (HGP) *via* vagal efferent signaling to the liver (55) and promotes negative energy balance by reducing food intake (56). Leptin binding in the ARC activates POMC neurons and inhibits AgRP neurons to reduce feeding, increase energy expenditure, and decrease HGP (57–59). The central action of these hormones plays an important role in the control of whole-body metabolism through signaling to peripheral organs. Insulin and leptin resistance are hallmarks of obesity, occurring following overnutrition and before the onset of major weight gain (60), and both are associated with mitochondrial dysfunction in the brain (61).

**Figure 1:**
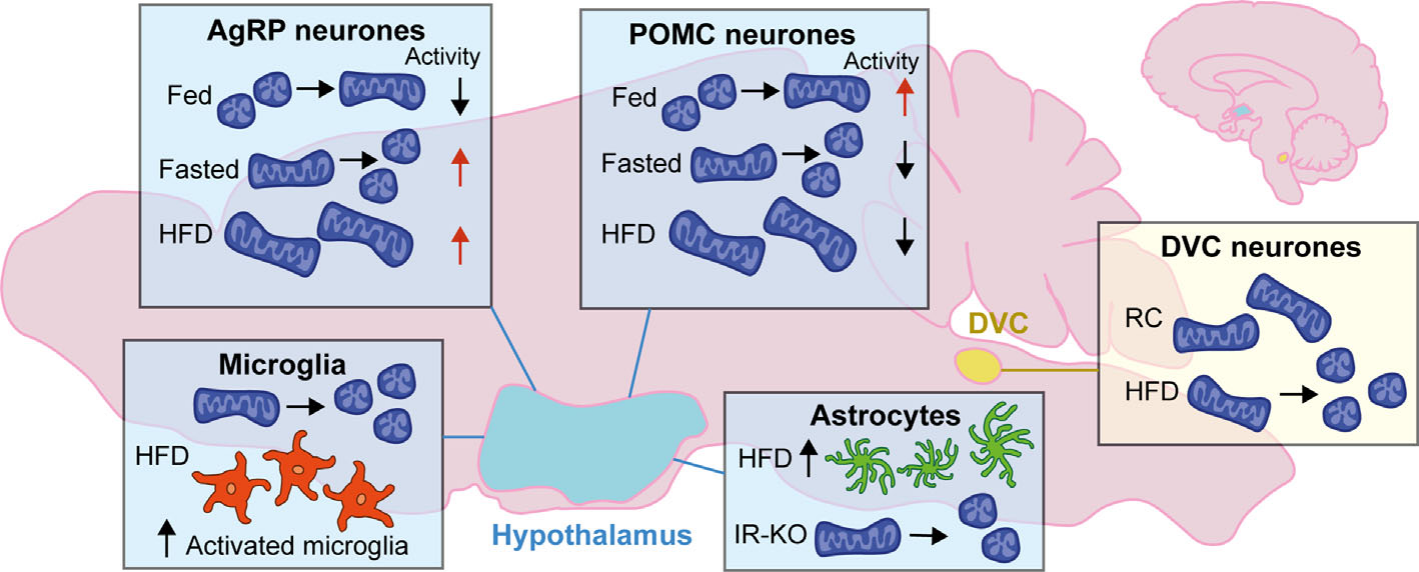
Changes to mitochondrial dynamics in response to diet in the hypothalamus and DVC: Mitochondrial dynamics in the brain change depending on energy status. In the hypothalamus, neurons favor mitochondrial fusion following feeding and fission when fasted. The activity of AgRP and POMC neurons are antagonistic; following feeding AgRP neurons decrease activity while POMC neuron activity is increased while in the fasted state it is the opposite. Following high-fat diet (HFD) mitochondria favor fusion and become enlarged; AgRP neurons are highly active, and POMC neuronal activity is decreased. In microglia of the hypothalamus mitochondria favor fission with HFD, and there is an increase in the number of activated microglia in the ARC. There is also an increase in the number of astrocytes in the hypothalamus with HFD, and knockout of the insulin receptor (IR-KO) in astrocytes led to favoring mitochondrial fission. In the DVC, HFD leads to mitochondrial fission when compared to regular chow (RC) fed rodents.

In hunger-promoting AgRP/NPY neurons, fasting increases mitochondrial fission whereas feeding and overnutrition promote fusion *via* MFN1 and MFN2 (62) (**Figure 1**). In a mouse model of diet-induced obesity (DIO), silent AgRP/NPY neurons presented with mitochondria that were increased in size and more elongated than those in wildtypes (62), demonstrating a role for mitochondrial fusion in AgRP neuronal response to overnutrition. Interfering with mitochondrial fusion in AgRP neurons by deletion of *Mfn1* or *Mfn2* resulted in altered mitochondria size and density, as well as some physiological phenotypes (62). Mice lacking *Mfn1* in AgRP neurons displayed no metabolic phenotypes on a regular chow diet; however, following HFD food intake and body weight were decreased in female mice, and AgRP neuronal firing rate was decreased in both males and females. *Mfn2* deletion in AgRP neurons did not alter the number of mitochondria but led to a decrease in body weight of females fed regular chow. *AgRP-Mfn2^−/−^* mice fed HFD gained less weight compared to wildtype mice. AgRP firing rate was decreased, and the number of mitochondria was increased due to failed fusion (62). Altogether these data indicate a critical role for mitochondrial fusion in AgRP neurons in the maintenance of metabolism and appropriate weight gain following overnutrition.

Mitochondrial remodeling enzymes are also implicated in anorexigenic POMC neuronal signaling. Following feeding, mitochondrial fusion occurs in POMC neurons; this is mediated by a reduction in activated DRP1 levels (63) (**Figure 1**). Deletion of *Mfn1* and *Mfn2* in POMC neurons leads to a strikingly different phenotype compared to AgRP/NPY-specific deletion of these key fusion proteins (62, 64, 65). MFN1 and MFN2 have non-redundant and distinct roles; MFN1 is more efficient at mediating the process of mitochondrial fission than MFN2 (66), whereas MFN2 is also involved in the establishment of mitochondrial-endoplasmic reticulum (ER) contacts (67). These differences are exemplified by discordant alterations in POMC neuron function following specific KO of either *Mfn1* (65) or *Mfn2* (64).

In DIO mice downregulation of *Mfn2* alters mitochondrial network dynamics, reduces mitochondria–ER contacts, and impairs intracellular Ca^2+^-handling (64, 68). Ablation of *Mfn1* in POMC neurons in mice led to defective mitochondrial architecture remodeling following feeding (65). Mice also presented with reduced insulin secretion by pancreatic *β*-cells after glucose challenge; this was recovered following administration of a sympathetic antagonist, suggesting a neural basis for the mechanism. Loss of *Mfn1* caused an increase in mitochondrial respiration and ROS production in fed, but not fasting, conditions compared to wildtype mice (65). Deletion of *Opa1* in the same neuronal population caused reduced mitochondrial fusion; however, neuronal activation, insulin secretin and hypothalamic ROS production remained unchanged (65). Selective ablation of *Mfn2* from POMC neurons in mice (*POMCMfn2KO*) caused leptin resistance, hyperphagia, and a reduction in energy expenditure and obesity (64). MFN2 is involved in tethering the ER to mitochondria (67), and deletion is known to cause ER stress (64, 69). Mitochondria–ER contacts were significantly reduced in *POMCMfn2KO* mice; ER stress markers were upregulated, and ROS levels were increased (64). Interestingly, antioxidant enzymes were increased in the hypothalamus of these mice, suggesting a potential compensatory mechanism to reduce excess ROS. When ER stress was relieved with the chemical chaperone 4-phenylbutyric acid (4-PBA) food intake and body weight were normalized in *POMCMfn2KO* mice, while there were no changes in control mice (64). Together these data highlight the role of mitochondrial fusion and ER stress in POMC neurons as regulators of central metabolic signaling and responsiveness to circulating satiety factors both centrally and in peripheral tissues. In further agreement for the role of mitochondrial fusion in POMC neuron function, the fission mediator DRP1 was found to be phosphorylated (Ser616) and active in silent POMC neurons (63). Inducible deletion of *Drp1* in POMC neurons led to increased neuronal activity, improved leptin sensitivity, and glucose metabolism, as well as increased hepatic glucose output (63). Mitochondria were increased in size, and ROS production was increased.

Anorexigenic POMC neurons are inhibited by increased mitochondrial fission leading to reduced energy expenditure and increased weight gain. Conversely, when fission is increased in orexigenic AgRP/NPY neurons it is beneficial in terms of weight gain, and increased caloric intake does not result in the same increase in body weight. HFD is associated with mitochondrial fusion in both POMC and AgRP neurons with opposing effects on neuronal activity; POMC neuronal activity decreases, while AgRP neuronal activity increases, leading to further increased food intake (62, 68) (**Figure 1**). Collectively, these data demonstrate the importance of mitochondrial dynamics in neuronal populations of the MBH and their role as essential regulators in the modulation of metabolism.

## Mitochondrial Dynamics and Whole-Body Metabolism in the DVC

More recently it was discovered that the DVC of the brainstem is involved in metabolic homeostasis and central response to insulin (70–72). The DVC consists of three regions: the dorsal motor nucleus (DMX), the nucleus tractus solitarius (NTS), and the AP. It is a site of integration, receiving signals from the peripheral organs and relaying them to the brain, while also relaying brain signals to the peripheral organs; this makes it a crucial region when studying neuronal control of physiological functions. Insulin in the DVC activates Erk1/2 rather than PI3K/Akt signaling as it does in the MBH (70). Infusion of insulin in the DVC of rodents lowered hepatic glucose production, and activation of K_ATP_ channels was required for insulin-Erk1/2 action (70).

HFD feeding in rodents induces insulin resistance (60), both in peripheral organs and in the brain (70, 73). After just three days of HFD infusion of insulin into the DVC fails to activate Erk1/2, where this central insulin resistance occurs prior to substantial weight gain and is bypassed by activation of K_ATP_ channels in the DVC by diazoxide (70). Furthermore, acute insulin infusion into the DVC decreased food intake in regular chow fed rats but failed to do so in HFD-fed rats (71). Upon overnutrition, mitochondria undergo Drp1-mediated fission in DVC neurons, thereby suggesting a role for mitochondrial dynamics in DVC insulin resistance (72) (**Figure 1**). Indeed, inhibition of DRP1 *via* infusion of MDIVI-1, which blocks DRP1 translocation from the cytosol to the mitochondria (74), in the DVC reversed the HFD-induced changes to mitochondrial dynamics and prevented the development of insulin resistance (72). DRP1 is regulated by post-transcriptional modifications including phosphorylation and SUMOylation (75); a decrease in phosphorylation of serine-637 increases DRP1 activity (76). When Drp1 activity was increased *via* delivery of a phospho-deficient mutant (Drp1-S637A) in the DVC of healthy regular chow fed rats, insulin resistance was induced (72). ER stress is necessary for Drp1-dependent mitochondrial fission to induce insulin resistance in the DVC. When the ER stress inhibitor 4-PBA was delivered to the DVC of HFD-fed rats, the ability of insulin to lower glucose production was restored (72). Together these data demonstrate the importance of Drp1-mediated mitochondrial fission and ER stress in the DVC in the development of insulin resistance following overnutrition and highlight the potential therapeutic targets in the treatment of obesity and T2D.

Studies into the role of mitochondrial dysfunction in the brainstem and the effect on metabolism are still relatively novel. It is yet to be elucidated whether disruption of mitofusins or OPA1 in the DVC can alter feeding behavior and HGP. While there is a clear role for mitochondrial dysfunction in the development of insulin resistance in the DVC, it is unknown as to whether it also contributes to leptin resistance. More research is therefore required to define the role of mitochondria in the DVC in the development of obesity and T2D.

## Energy Homeostasis and Mitochondrial Dynamics in Glial Cells

Mitochondrial function is crucial to all cell types, and disruption in glial cells of the brain is associated with metabolic disorders (14). Astrocytes and microglia produce cytokines that drive inflammation and thus are mediators of the response to overnutrition and are involved in the development of obesity and T2D (77). Astrocytes and microglia may develop a reactive phenotype in response to injury or disease, also referred to as gliosis, which can be measured by changes in morphology and protein expression. Acute gliosis is protective and beneficial to neurons, but chronic activation can be detrimental. Increased gliosis has been measured in the hypothalamus of obese humans and in rodent models of obesity (77) (**Figure 1**).

Astrocytes regulate essential aspects of CNS function and brain energy metabolism (78). Their physical proximity to blood vessels and ability to transport nutrients mean they are directly affected by nutrient excess. Astrocytes exhibit a high rate of oxidative metabolism (79) and can switch between oxidative phosphorylation and glycolysis for their energy supply depending on local energy needs (80, 81). Inflammation alters mitochondrial dynamics in astrocytes where Drp1 is phosphorylated leading to increased fission, ROS production, and reduced respiratory capacity (82). Thus, the low-grade chronic inflammation associated with obesity is likely to mediate some of its effect *via* astrocytes.

Mice fed HFD for 10 days had an increased number of astrocytes in the ARC of the hypothalamus (83) (**Figure 1**). Inhibition of NF-*κ*B in astrocytes prevented acute HFD-induced astrocyte activation and reduced food intake (84). DREADD-mediated activation of astrocytes in the DVC of mice reduced dark-phase feeding and reduced refeeding after overnight fasting (12). Furthermore, activation of DVC astrocytes induced signaling in the lateral parabrachial nucleus (12), another region involved in appetite regulation and glucose homeostasis (85–87). Conditional deletion of *Mfn2* in astrocytes of mice led to a reduction in mitochondria–ER contact sites within astrocytes and associated disruption of calcium buffering; however, this study did not measure changes related to feeding (88). Targeted postnatal ablation of the insulin receptor in astrocytes of mouse hypothalamus did not alter body weight compared to control mice in either regular chow or high-fat, high-sugar diet, but mitochondria within astrocytes were fewer and smaller in response to elevated blood glucose levels (16) (**Figure 1**). HFD promoted loss of synapses on POMC neurons in the ARC due to reactive gliosis where glial ensheathment of the soma was increased (89). This altered synaptic organization led to increased POMC tone and a decrease in excitatory connections onto neighboring neuropeptide Y (NPY) cells.

These data highlight the role of astrocyte–neuronal crosstalk in energy homeostasis and how altered astrocyte function can affect mitochondrial networks in neurons. As yet, more work is needed to elucidate the role of mitochondrial dynamics and dysfunction in astrocytes and how these relate to whole body metabolism, feeding behavior, and hormone sensitivity in the hypothalamus and DVC.

Microglia are the immune cells of the central nervous system, acting to protect from invaders and orchestrating the inflammatory response. Three days of HFD in rats led to an increased number of microglia in the ARC compared to regular chow fed controls along with increased expression of inflammatory mediators including IL-6 and SOCS3 (77) (**Figure 1**). Central modulation of inflammation by inhibition of sodium-glucose transporter 2 (SLGT2) in obese insulin-resistant mice blocks microglia activation in the hypothalamus and restores insulin sensitivity (90, 91). Microglia are crucial in the development of obesity and T2D as they initiate the inflammatory response following overnutrition and increased circulating free fatty acids (13).

Recently, mitochondrial dynamics in microglia have begun to be studied, primarily in the hypothalamus. Three days of HFD in mice led to an increase in activated DRP1 in hypothalamic microglia, and mitochondria were decreased in size and increased in number (15) (**Figure 1**). High-fat feeding also increased expression of the mitochondrial protein UCP2 (Uncoupling protein 2) in microglia (15). UCP2 plays a critical role in mitochondrial function, including control of ROS generation (92, 93) and is highly expressed in activated microglia (94). Selective deletion of *Ucp2* in microglia of adult mice (*Ucp2* ^MGKO^) prevented changes to mitochondrial dynamics induced by HFD (15); mitochondria did not change in size, number or coverage. *Ucp2* ^MGKO^ mice did not present with activated microglia in the ARC following HFD, and cytokine levels were reduced. Furthermore, *Ucp2* ^MGKO^ mice were protected from diet-induced obesity; their food intake was reduced, energy expenditure increased, and insulin sensitivity improved compared to control mice on HFD. It has been previously reported that HFD alters POMC synaptic input organization *via* reactive gliosis (89), *Ucp2* ^MGKO^ mice were protected, and synapses onto POMC cell bodies were increased along with increased POMC activation (15). This suggests that activated microglia may be upstream controllers of synaptic plasticity and astrogliosis in response to overfeeding and present a potential therapeutic avenue for alleviating obesity. As yet the effects of other mitochondrial proteins in the microglia have not been studied nor has the role of microglia in the DVC in DIO models.

## Mitochondrial Dynamics and Glucose Metabolism in the Brain

Glucose is the main fuel utilized by the brain where brain glucose metabolism also provides the energy and precursors for the biosynthesis of neurotransmitters (95); therefore, tight regulation of glucose homeostasis is essential to maintain blood glucose level for optimal brain and CNS function. Glucose is a large polar molecule which cannot easily pass the lipid cell membrane by simple diffusion; therefore, entry of glucose into the CNS and its cells are achieved by transport proteins known as glucose transporters (96, 97). Glucose transporters are comprised of two main types which include sodium-glucose linked transporters (SGLTs) which symport glucose across the cell membrane with Na^+^ along a sodium gradient, and sodium-independent transporters (GLUTs), which transport glucose across the plasma membrane by a facilitated diffusion mechanism (96, 97). GLUT-1 is expressed by endothelial cells at the BBB and is the predominant transporter which is responsible for the movement of glucose into the brain (98), where it is then taken up by neurons and astrocytes which predominantly express GLUT3 and GLUT1, respectively (99–101). While nearly all of the six identified SGLTs, apart from SGLT5, have been shown to be expressed in the brain; the distribution of SGLT expression and their contribution to glucose uptake differ strongly across the brain (102, 103). SGLT1 is the most studied of this family, where reports have shown that SGLT1 accounts for 80% of SGLT-mediated glucose uptake in the midbrain (102) and that 45% of GE-neurons tested in the ventromedial hypothalamus (VMH) are activated by an SGLT1-specific ligand (104).

Following entry to the CNS, the MBH and DVC sense glucose, circulating hormones including insulin, and nutrient availability to control CNS glucose levels and glucose metabolism by alterations in feeding behavior, HGP, and peripheral glucose uptake and utilization (70, 105). For some time, the intracellular mechanisms underlying the ability of these cells to sense, respond to, and influence changes in glucose homeostasis have been unclear. However, emerging research is beginning to show that the dynamic nature of mitochondria, their energy sensing properties, and their ability to undergo fusion and fission, may be critical for the ability of these cells to contribute to the central regulation of metabolism.

## Glucose Sensing in the Hypothalamus

**Figure 2:**
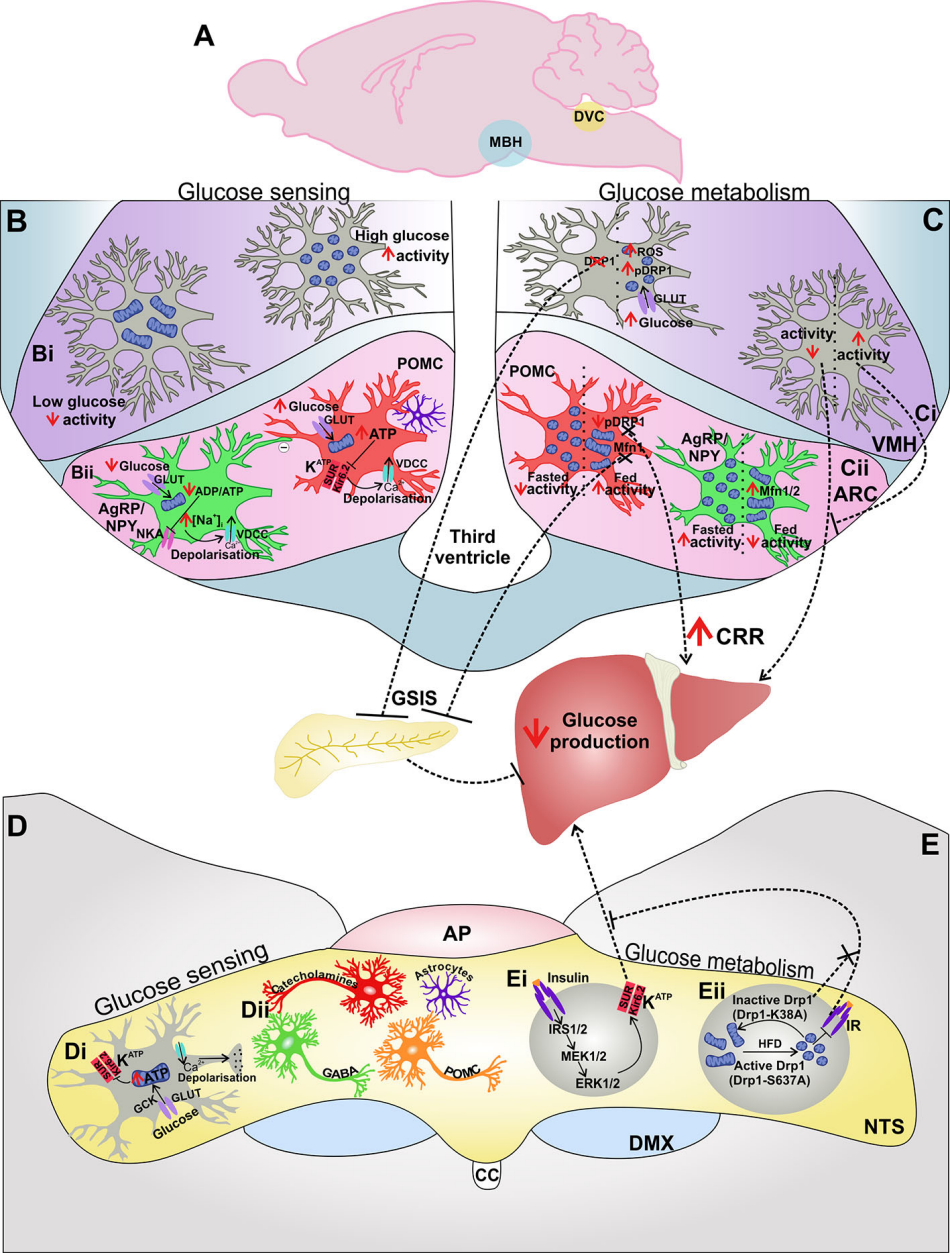
The MBH and DVC are involved in central glucose sensing and control of glucose metabolism: The MBH and DVC are distinct areas which are involved in both glucose sensing and glucose metabolism **(A)**. Glucose sensing cells in the MBH **(B)** are present in both the VMH **(Bi)** and ARC **(Bii)**. In the VMH increased glucose leads to mitochondrial fission and increased neuronal activity **(Bii)**. In the ARC, glucose-inhibited (GI) AgRP and glucose-excited (GE) POMC neurons utilize the Na^+^/K^+^-ATPase (NKA) or K_ATP_ channels in order to couple extracellular glucose levels and energy status with neuronal activity **(Bii)**. Astrocytes also influence the activity of POMC neurons in response to changes in glucose availability **(Bii)**. The MBH also influences glucose metabolism **(C)**, where neuronal activity and increased mitochondrial fission and neuronal activity in VMH neurons induce glucose-stimulated insulin secretion (GSIS) from the pancreas and decrease glucose production **(Ci)**. Mitochondrial dynamics in AgRP and POMC neurons in the ARC influence neuronal activity of these neurons in response to fasted *vs* fed states **(Cii)**. Inhibition of mitochondrial fusion in POMC neurons inhibits GSIS **(Cii)**. The DVC is also able to sense glucose **(D)**, utilizing KATP channels to transduce changes in extracellular glucose levels and therefore energy availability to neuronal activity **(Di)**. Various cells have been implicated in glucose sensing in the DVC, including catecholaminergic-, GABAergic-, POMC neurons, and astrocytes **(Dii)**. The DVC also influences glucose metabolism **(E)** by insulin signaling mediated ERK1/2 activation of K_ATP_ which decreases glucose production **(Ei)**. Mitochondrial fission inhibits insulin receptor signaling in the DVC and impairs DVC control of glucose homeostasis by inhibiting reduced HGP **(Eii)**. Abbreviations: CRR, counter-regulatory response; GCK, glucokinase; GLUT, glucose transporter.

The hypothalamus contains several nuclei which are involved in the central control of metabolism, including the ARC, ventromedial, paraventricular (PVN), and dorsomedial hypothalamus (DMH) (106) (**Figures 2A–C**). Specialized glucose sensing cells are present within hypothalamic nuclei which integrate and control neuroendocrine signaling and function and play an essential role in the regulation of systemic glucose levels, acting as a critical site for counter-regulatory mechanisms in response to both hypo- and hyperglycemia (106–108).

Neurons in the ARC control systemic glucose levels by neural regulation of HGP (108), whereas glucose sensing neurons in the VMH prevent severe hypoglycemia by activating the counter-regulatory response (CRR) which engages several defensive mechanisms to restore glucose homeostasis. Glucose sensing neurons detect changes in glucose levels either above or below 2.5 mM (109) and respond to extracellular glucose concentration by changing their firing rate; glucose-excited (GE) cells increase their rate of neuronal firing, whereas glucose-inhibited (GI) cells decrease their firing when exposed to a rise in extracellular glucose (107, 110). Neuronal glucose responsiveness is associated with the activity of K_ATP_ channels which couple the energy status of the cell and electrical activity, closing the channel and triggering depolarization in a high ATP/ADP ratio and opening the channel causing hyperpolarization in a low ATP/ADP ratio (111, 112) (**Figure 2Bii**). In response to changing energy demand and supply, mitochondria adapt by changing the capacity and/or efficiency of ATP production which will therefore influence the activity of K_ATP_ channels and neuronal activation of glucose sensitive cells.

Glucose transporters, both GLUTs and SGLTs, have also been implicated in hypothalamic glucose sensing. GLUT2 is required for glucose sensing in pancreatic *β*-cells and evidence suggests that it may also perform a similar role in the brain. For example, GLUT2 ShRNA induced impaired GLUT2 function in the ARC, blunted the increase in insulin secretion following administration of a glucose bolus and diminished glucoprivic feeding behavior (113, 114). Furthermore, transgenic mouse lines with either brain-specific GLUT2 deletion (115) or inhibition of GLUT2-mediated extracellular glucose detection (116, 117) also support the role of GLUT2 in hypothalamic glucose sensing and control of food intake. Another member of the GLUT family, GLUT4, has been implicated in glucose sensing, particularly as it is expressed in 57% of GE neurons and 63% of GI neurons in the VMH (118). Indeed, it has been shown that the CRR to hypoglycemia is impaired in mice following brain-specific KO of GLUT4 (119). Conversely, a reduction in hypothalamic SGLT1 expression using ShRNA was found to improve the CRR to hypoglycemia, leading to enhanced HGP, showing that SGLT1 in the VMH is also involved in glucose sensing and regulation of glucose homeostasis (120). It would therefore seem that glucose transporters also contribute to the glucose sensing machinery in the hypothalamus, and further work is required to determine how glucose availability by glucose transport, glucose-sensing by the transporters themselves, and ATP production by mitochondria may be linked in the act of hypothalamic glucose sensing.

Astrocytes also play a crucial role in regulation of glucose homeostasis, modulating glucose availability, and therefore the levels of brain glucose metabolism, *via* control of glucose transport across the BBB by GLUT-1, which is highly expressed in astrocytes (16, 121). This is exemplified by the finding that IR-KO in GFAP^+^ astrocytes also resulted in reduced expression of GLUT1 and a concomitant reduction in CSF glucose concentration (16). As well as responding to insulin and leptin (16, 122), astrocytes also express receptors for the hormone glucagon-like peptide 1 (GLP-1) (123–125). Activation of GLP-1 receptors in astrocytes of the hypothalamus inhibited astrocytic glucose uptake and promoted fatty acid utilization (126). Furthermore, knockout of GLP-1 receptors from hypothalamic astrocytes abolished alterations in mitochondrial dynamics associated with acute glucose exposure *ex vivo* (126). In the ARC, ablation of astrocytic GLP-1 receptors in mice improved glucose availability and peripheral glucose metabolism (126), further highlighting the role for astrocytes in control of glucose homeostasis.

AgRP and POMC neurons of the ARC have opposing effects on glucose metabolism (127) (**Figure 2Cii**). The mechanisms which allow these two cell types to respond to, and influence, whole body energy status have been elusive. However, mitochondrial dynamics in both AgRP/NPY and POMC neurons have recently been shown to be regulated by nutrition in an opposing manner in these two cell types (62, 64) (**Figure 2Cii**). Changes in mitochondrial dynamics are involved in how these cells respond to glucose levels by altering neuronal activation and thereby influence systemic glucose metabolism.

## Glucose Sensing and POMC Neurons

Neuronal activity of anorexigenic POMC is influenced by extracellular glucose levels, where a subset of POMC neurons are GE (64, 128, 129). In the postprandial state, when extracellular glucose rises, K_ATP_ channels in POMC neurons close, leading to depolarization and increased neuronal firing (128, 129) (**Figure 2Bii**). GE POMC neurons exhibit a 2.26 +/− 0.23-fold faster firing rate in 5 mM glucose compared to 3 mM glucose (129). Transgenic expression of a mutant Kir6.2 subunit in POMC neurons, which prevents ATP-mediated closure of K_ATP_ channels, resulted in a failure of POMC neurons to increase their firing rate in response to high glucose levels and an impairment in the whole-body response to systemic glucose load (129). K_ATP_ channel function is therefore required for activation of POMC neurons by glucose which contributes to the overall control of glucose homeostasis. Furthermore, although POMC neurons are clearly activated by glucose (129), it has been argued that no study has yet shown that glucose directly modulates the activity of these neurons (109). Instead, it has been hypothesized that glucose modulates synaptic remodeling to alter the frequency of excitatory postsynaptic currents onto POMC neurons (130), perhaps by retraction of glial coverage (131) (**Figure 2Bii**).

HFD decreases spontaneous activity and hyperpolarizes the membrane potential of POMC neurons (68, 132). Glucose responsiveness by POMC neurons is also abolished in obese mice following a HFD, which is linked to an increase in the mitochondrial protein UCP2. In pancreatic *β*-cells, UCP2 negatively controls glucose sensing by mediating proton leak across the inner mitochondrial membrane, decreasing the yield of ATP from glucose (133). In POMC neurons, UCP2 is also involved in diet-induced loss of glucose sensing, perhaps by decreasing ATP production by POMC neurons (129). Genetic knockdown of *Ucp2* can prevent diet induced obesity and restore insulin sensitivity in leptin deficient *ob/ob* mice (129, 133). These results highlight the importance of mitochondria in glucose sensing and illustrate that disruption of these processes following excessive energy intake may be an important pathogenic component in obesity associated loss of glucose and insulin sensitivity in T2D.

Astrocytes in the ARC also play a role in glucose metabolism by influencing POMC neuronal activity (16) (**Figure 2Bii**). Neurons make approximately 13% of the brains ATP from glucose which is transported across the BBB; the remaining 87% is generated by glial cells (134). Therefore, together, astrocytes and neurons form a metabolic unit that is able to monitor circulating nutrients to alter food intake and energy homeostasis.

Inducible postnatal deletion of insulin receptor specifically in GFAP^+^ astrocytes (GFAP-IR-KO) caused a loss insulin signaling activation of Akt in astrocytes with no effect on peripheral insulin signaling. Astrocyte-specific loss of insulin receptors decreased hypothalamic glucose sensing as GFAP-IR-KO blunted the activation of POMC neurons to cause an increase in systemic glucose levels (16). The mitochondria play a critical role in neuronal responses to fluctuations in nutrient availability by inducing alterations in the mitochondrial network of nutrient sensing neurons, including changes in mitochondrial morphology, mitochondrial density, and coverage, and the number of contacts between mitochondria and the ER (135, 136). For example, POMC neurons in GFAP-IR-KO mice exhibit decreased mitochondrial density and mitochondrial coverage in response to elevated glucose levels. Furthermore, in response to glucose administration, GFAP-IR-KO mice had higher numbers of ER–mitochondrial contacts in POMC neurons compared to WT mice (16). These results illustrate how insulin signaling in astrocytes is required for appropriate POMC neuronal function in response to cellular metabolic needs and show how astrocyte–neuronal crosstalk in the ARC influences metabolic homeostasis.

## Mitochondrial Dynamics in POMC Neurons Associated With Glucose Metabolism

The mitochondria also act as energy sensors in anorexigenic POMC neurons in order to balance energy demand and nutrient supply, where food deprivation results in a decrease in mitochondrial density and coverage in POMC neurons (62) (**Figure 2Cii**). Conversely, mitochondria number is highest, and mitochondrial length and branching are decreased in POMC neurons in fed and diet-induced obese mice when compared to fasted or leptin deficient *ob/ob* animals (64, 137) (**Figure 2Cii**).

MFN1 has recently emerged as a nutrient sensor in POMC neurons which influences whole-body glucose metabolism as it plays a key role in the central control of insulin release. Structural and functional alterations in mitochondria were found in POMC neurons with selective deletion of *Mfn1* (64). Furthermore, POMC *Mfn1* KO impaired central glucose sensing, perturbed glucose-stimulated insulin secretion (GSIS), and altered glucose metabolism (65) (**Figure 2Cii**), indicating the importance of mitochondrial fusion in POMC-mediated glucose homeostasis.

During normal metabolism of oxygen by mitochondria, ROS are formed as a natural by-product (138). Release of ROS from POMC neurons is required for POMC neuronal activation in response to rising glucose levels (139, 140). ROS activate POMC neurons by modulation of membrane potential and neuronal firing to promote satiety and increase energy expenditure, for example ROS levels are significantly higher in POMC neurons following HFD feeding (137). Mitochondrial morphology is also associated with ROS production and energy status (141). MFN1 appears to be important for this process in POMC neurons, as *Mfn1*-specific KO in these cells results in increased mitochondrial respiration and ROS production in response to high glucose (65). Furthermore, transient excessive ROS production in POMC neurons leads to defective GSIS in *POMCMfn1KO* mice as central administration of two chemically distinct ROS scavengers normalized GSIS in these animals (65). Loss of MFN1 in POMC neurons therefore impacts mitochondrial flexibility and interferes with POMC neuronal responses, hindering glucose sensing and sympathetic nervous system driven peripheral responses which modulate glucose metabolism such as insulin release.

The fusogenic protein MFN2 is also important for mitochondria–ER tethering (67). HFD results in a downregulation of *Mfn2* expression, increased mitochondrial density, and decreased mitochondria–ER contacts in POMC neurons (64), illustrating that MFN2 is involved in metabolic alterations following diet-induced obesity and so may be involved in the central regulation of energy balance.

Whilst liver glucose output and expression of gluconeogenic genes were normal in POMCMfn1 KO loss of *Mfn2* resulted in enhanced HGP. These changes were a result of alterations in mitochondrial morphology, reduced numbers of mitochondria–ER contacts, and increased ER stress in POMC neurons. ER contact is needed for Ca^2+^ uptake in the mitochondria (67). Diet-induced obesity has been shown to reduce mitochondrial Ca^2+^ accumulation capacity in POMC neurons, leading to greater levels of free intracellular Ca^2+^ which contributes in part to a reduction in POMC neuronal excitability by activation of hyperpolarizing Ca^2+^-activated K^+^ channels (68). It could therefore be hypothesized that reduced mitochondria–ER contact in *POMCMfn2KO* mice also results in alterations in POMC neuronal activity by changes in the levels of intracellular Ca^2+^; however, this remains to be determined.

These results show that like MFN1, MFN2 is also required for POMC neuronal function and systemic energy balance as it is critical for mitochondria–ER interaction (64). *POMCMfn1KO* was found to alter GSIS by increasing POMC ROS levels, *Mfn2* deletion also increased ROS production, illustrating that there may also be some shared roles for MFN1 and MFN2 in POMC neuron function. Deletion of the other fusogenic protein *Opa1* in POMC neurons failed to produce the same phenotypic features of either *Mfn1* or *Mfn2* KO; these data therefore illustrate the importance of *Mfn*-specific mitochondrial functions in POMC neurons for the regulation of whole-body energy/glucose homeostasis.

The importance of mitochondrial fusion in the control of glucose sensing in POMC neurons is also clear when examining the role of the fission protein DRP1. Inducible deletion of Drp1 in mature POMC neurons improves their glucose responsiveness, as measured by stronger POMC neuronal inhibition in response to decreased extracellular glucose levels (63). This was paired with a significant improvement in peripheral glucose tolerance, an increase in food intake in response to glucoprivation, and enhanced hepatic counter-regulatory responses to hypoglycemia (63) (**Figure 2Cii**). Glucose metabolism increases levels of POMC ROS and activation of the nuclear receptor Peroxisome proliferator-activated receptor gamma (PPAR*γ*) which increases neuronal firing (137). This crucial process is also linked to Drp1-induced mitochondrial fission, as following exposure to rising glucose load, POMC neurons with Drp1 deletion showed increased levels of ROS and PPAR*γ* protein levels alongside greater neuronal activation compared to control (63). Increased ROS and PPAR*γ* levels in animals with POMC Drp1 deletion resulted in greater POMC neuronal activity in response to metabolic shifts by altering K_ATP_ channel expression (63). These results show that DRP1-mediated mitochondrial fission plays a negative role in POMC neuronal responses to glucose load and further highlight that mitochondrial dynamism is critical for the central regulation of glucose metabolism.

## Glucose Sensing in AgRP Neurons

Much like in anorexigenic POMC neurons of the ARC, glucose is also an important regulator of AgRP/NPY neuronal activity. For example, I.P or ICV injection of glucose in rats produces a 1.8 mM rise in circulating glucose and a 30–60% reduction in NPY and AgRP mRNA in the ARC at 30- and 60-min post injection. After 90 min circulating glucose levels decline, and the suppressive effect of glucose administration is lost, while NPY and AgRP levels increase by 50% (142). These results illustrate that AgRP/NPY neurons are sensitive to changes in circulating glucose levels and respond accordingly to modulate energy balance by altering levels of AgRP and NPY which influence energy intake and expenditure (143–145). Half of AgRP/NPY expressing neurons of the ARC are inhibited by physiological brain glucose levels (146). In response to decreased glucose levels AgRP/NPY neurons are activated, leading to increased *c-fos* expression, enhanced hypothalamic NPY release, depolarization and greater neuronal firing, and a concurrent drive to feed when there is an energy deficit (147–149). A recent study using NPY-deficient mice found that NPY is necessary for the immediate feeding behavior and acute glucose homeostasis functions which are governed by AgRP/NPY neuron activation (150).

While the exact mechanism by which decreased glucose induces depolarization in AgRP/NPY neurons is yet to be determined, the Na^+^/K^+^-ATPase (NKA) pump has been evidenced as one of the molecules which converts low glucose levels to changes in neuronal excitability in glucose sensitive cells (**Figure 2Bii**). Fasting glucose levels suppress the enzymatic activity of NKA by decreasing ATP-hydrolyzing activity by half and ATP substrate availability by a third (151). These changes lead to depolarization of GI AgRP/NPY neurons *via* an increase in the level of intracellular Na^+^, illustrating how suppression of NKA by decreased ATP availability in negative energy states is tightly linked to AgRP/NPY activation (152) (**Figure 2Bii**). In contrast, changes in the activity of the energy sensor AMP-activated protein kinase (AMPK) has been shown to be required for hyperpolarization and therefore inactivity of GI neurons following exposure to glucose (152, 153). AgRP/NPY neurons also play a fundamental role in mediating the central inhibitory effect of insulin on HGP (55, 154), thereby illustrating the dual role AgRP/NPY neurons play in modulating whole body energy balance by responding to both insulin and glucose.

## Mitochondrial Dynamics in AgRP/NPY Neurons Associated With Glucose Metabolism

As in POMC neurons, mitochondria also act as energy sensors in AgRP/NPY neurons; however, the electrical and mitochondrial morphological responses of AgRP/NPY neurons to nutrition oppose those of POMC neurons. By sensing changes in extracellular glucose levels neuronal activity of AgRP/NPY neurons is increased during periods of fasting or negative energy balance (155, 156). There are also parallel changes in mitochondrial morphology in these neurons; for example, in fasted states mitochondria in AgRP/NPY are decreased in size, and mitochondrial density is increased (62, 157) (**Figure 2Cii**). These changes are indicative of increased levels of mitochondrial fission and suggest that fission may be a requirement for the activation of orexigenic AgRP/NPY in times of negative energy balance. Indeed, expression of the key fission protein Drp1 has been found in AgRP/NPY neurons (62). However, as yet, no studies have examined the effect of Drp1 KO in AgRP/NPY neurons upon glucose sensing and metabolism. It would therefore be interesting to see whether impairment of Drp1-dependent mitochondrial fission decreases glucose responsiveness and prevents increased neuronal activity in response to negative energy balance in AgRP/NPY neurons, particularly as Drp1 KO improved neuronal glucose responsiveness in POMC neurons (63).

Paradoxically, in HFD-fed mice AgRP neurons are highly active (137) and exert a greater inhibitory tone upon anorexigenic POMC neurons (158). AgRP/NPY neurons are normally GI; however, when exposed to HFD, these neurons show an increase in electrical activity in response to elevated levels of glucose (62). Mitochondria in AgRP neurons following HFD feeding were found to be significantly larger; however, mitochondrial density was decreased, indicating that nutrient excess in HFD leads to increased fusion in AgRP neurons (62). Deletion of *Mfn1* or *Mfn2* in AgRP/NPY neurons prevents fusion and impairs neuronal firing frequency in diet-induced obesity (62), illustrating that the induction of mitochondrial fusion is required for the paradoxical neuronal activation of AgRP/NPY neurons following exposure to HFD (**Figure 2Cii**). Impaired neuronal activity of *Mfn*-deficient AgRP/NPY neurons was a result of altered intracellular ATP levels as cell-selective administration of ATP restored AgRP/NPY neuronal activity (62). *Mfn2-KO* mice fed HFD also had improved glucose tolerance, demonstrating the importance of mitochondria in whole body metabolic responses to positive energy balance. Such results therefore show how mitochondrial fission and fusion in AgRP/NPY neurons are dynamically regulated by nutritional and metabolic status and contribute to whole-body energy metabolism.

## Glucose Sensing in the Ventromedial Hypothalamus

The VMH also contains glucose sensing neurons and is another key area which is involved in the control of glucose homeostasis (118, 159, 160) (**Figure 2Bi**). Much like in the ARC, recent work has also illustrated that DRP1-mediated mitochondrial fission is essential for glucose sensing in the VMH and is therefore involved in how the VMH regulates systemic glucose metabolism (161–163) (**Figure 2Ci**). However, unlike in POMC neurons, Drp1 positively regulates glucose responsiveness of VMH neurons (161, 162).

Systemic administration of glucose decreased mitochondrial size and increased mitochondrial density without affecting total mitochondrial area in VMH neurons which is suggestive of glucose-induced mitochondrial fission (162) (**Figure 2Ci**). Indeed, the ratio of active phosphorylated DRP1 to non-phosphorylated DRP1 was significantly greater in VMH neurons following glucose infusion, confirming glucose-induced mitochondrial fission in these cells (162) (**Figure 2Ci**). Increases in extracellular glucose induce an increase in DRP1 translocation to mitochondria in the VMH; inhibition of this process blocks mitochondrial ROS production in response to glucose and impairs hypothalamic glucose sensing induced insulin secretion. Mitochondrial ROS production by DRP1-mediated mitochondrial fission in the VMH is also required for the satiation effect of intracarotid glucose infusion (161) (**Figure 2Ci**).

Impairment of hypothalamic glucose sensing and central GSIS has been described in animals fed a hypercaloric high-fat high-sugar (HFHS) diet for 3 weeks (163). HFHS-fed animals failed to increase mitochondrial ROS levels in response to an increase in glucose, where inhibition of mitochondria by rotenone treatment restored both ROS levels and hypothalamic glucose sensing induced insulin secretion in HFHS-fed animals. These HFHS diet-induced features were associated with a disruption in translocation of DRP1 to mitochondria in the hypothalamus in response to CNS hyperglycemia, further illustrating that DRP1-mediated mitochondrial fission and ROS production are important for regulating glucose metabolism by the VMH and can be impaired by caloric excess (161, 163).

Activation of DRP1 in the VMH is dependent upon the mitochondrial protein UCP2, as phosphorylation of DRP1 and therefore glucose-mediated mitochondrial fission, was absent in *Ucp2* KO mice (162). UCP2 has also been shown to modulate neuronal activity in part by controlling ROS production (164). UCP2 negatively regulates mitochondrial ROS production, where ROS levels in VMH neurons are involved in mediating GSIS in response to systemic glucose load (137, 161, 162). VMH neurons with *Ucp2* KO responded to glucose administration with an increased level of ROS compared to control animals; selective re-expression of UCP2 in the VMH restored ROS levels, illustrating that UCP2 enables VMH neuronal activation in response to rising glucose by buffering ROS. *Ucp2* KO animals were also glucose intolerant and exhibited reduced insulin sensitivity; these changes were also restored by re-expression of UCP2 in VMH neurons (162). Such results illustrate how UCP2 influences mitochondrial dynamics and neuronal activity in the VMH which are crucial for the regulation of glucose metabolism.

In conclusion mitochondrial dynamism in hypothalamic nuclei such as the ARC and the VMH is essential for the control of glucose homeostasis and the maintenance of energy balance (**Figure 2C**). Mitochondria are unique energy sensors, where alterations in mitochondrial products such as ATP, UCP2, or ROS influence the neuronal activity of glucose sensing cells in the hypothalamus, which in turn influences energy balance.

## Glucose Sensing and Mitochondrial Dynamics in the DVC

Although much of the work regarding the CNS control of metabolism and energy balance has concentrated on the hypothalamus, the DVC of the brainstem is the first site for the sensing and integration of peripheral neurochemical and humoral signals which communicate energy status to the brain (70, 165). Much like in the hypothalamus, glucose responsive cells are present in all three nuclei of the DVC (166); the DVC is therefore able to regulate glucose homeostasis by the CRR and is also able to influence HGP *via* DVC-hepatic vagal efferent neural circuitry (71, 167) (**Figures 2D, E**). The NTS also contains neurons that are activated by gastric distension; this acts as a postprandial satiety signal which reaches the NTS *via* vagal afferent fibers from the gut (168, 169).

The NTS is anatomically well situated to be influenced by dynamic changes in plasma glucose concentrations due its proximity to the AP, which is a circumventricular organ, and the presence of fenestrated capillaries within the NTS (170). It has long been known that the NTS contains cells which are gluco-responsive (166, 171). A subset of NTS neurons show depolarizing or hyperpolarizing responses to an increase or decrease in extracellular glucose concentration and are also involved in the CRR to hypoglycemia (172–176). NTS neurons respond to increased levels of glucose in a similar way to those in the hypothalamus which are mechanistically similar to pancreatic *β*-cells. These cells employ glucokinase (GCK) to monitor changes in glucose availability and mediate cellular utilization of glucose, GCK therefore links increased glucose to changes in membrane potential which occur *via* glucokinase-dependent modulation of K_ATP_ channels (172, 175, 177) (**Figure 2Di**). For example, K_ATP_ antagonists blunt the responsiveness of these NTS neurons to increased glucose (175, 177). It has also been shown that K_ATP_ channels of NTS neurons which respond to reductions in extracellular glucose levels, *i.e.* GI NTS neurons, are blocked by hyperglycemia and so blunt the neuronal response to low glucose levels (172). Therefore, in response to hyperglycemia, these GI NTS neurons lose the ability to respond to fast reductions in glucose, where pathological prolonged hyperglycemia such as in diabetes, may negatively impact the energy sensing abilities of K_ATP_ channels in the NTS (172).

There have been several neuronal types which have been identified as NTS glucose sensing neuronal candidates which play a role in regulating glucose metabolism. These include GLUT2^+^ GABAergic neurons which control vagal output and glucagon secretion (174, 177, 178), catecholaminergic neurons which mediate hypoglycemic hunger (179, 180), and NTS POMC neurons which are involved in short-term changes in feeding control (181, 182) (**Figure 2Dii**). Astrocytes in the NTS have also been shown to sense glucose and contribute to whole body energy balance by regulation of the hypoglycemia-induced counter-regulatory response (12, 183, 184) (**Figure 2Dii**). While much work has illustrated that mitochondria and mitochondrial dynamics are essential for the hypothalamic control of glucose homeostasis and that the NTS also influences energy balance by glucose sensing, how mitochondria are involved in these processes in the DVC is currently unknown.

The NTS is also able to sense insulin and responds to acute insulin by lowering HGP and regulating food intake and body weight *via* an insulin-ERK1/2 mediated signaling cascade which activates K_ATP_ channels in the DVC (70, 71) (**Figure 2Ei**). Activation of K_ATP_ channels is sufficient to induce a reduction in HGP and highlight the energy sensing abilities of K_ATP_ in the DVC which therefore influence whole body glucose metabolism (70). HFD feeding, or adenoviral-mediated constitutive activation of DRP1 (Drp1-S637A), results in a loss of the glucoregulatory effect of DVC insulin infusion (70, 72), where adenoviral-mediated molecular inhibition of DRP1-mediated mitochondrial fission by expression of a dominant negative form of DRP1 (Drp1-K38A) was found to restore the glucoregulatory effect of DVC insulin (72) (**Figure 2Eii**). These HFD-induced alterations in the mitochondria and insulin sensitivity in the DVC therefore require increased DRP1-dependent mitochondrial fission, where a HFD-fed phenotype could be recapitulated by adenoviral expression of a constitutively active form of DRP1, indicating that diet-induced changes in mitochondrial dynamism negatively affect central insulin sensitivity and HGP (**Figure 2Eii**). In summary, these results show that mitochondria in the DVC are essential for changes in peripheral glucose metabolism in response to DVC insulin sensing and illustrate how the DVC is a key area in the control of glucose homeostasis.

## Mitochondrial Dynamics Elsewhere in the Brain

While this review has focused on the role of mitochondrial dynamics in the hypothalamus and DVC in metabolism, the influence of mitochondria on CNS function throughout the brain has been studied. Mitochondrial dynamics are involved in a number of neurodegenerative conditions including Alzheimer’s disease, Parkinson’s disease, Huntington’s disease, and amyotrophic lateral sclerosis (ALS) [reviewed in (185–188)]. Indeed, obesity and diabetes are known to increase the risk of dementia (189, 190). Brain mitochondrial dysfunction in obesity and diabetes is associated with inflammation, ER stress, oxidative stress, all of which can exacerbate neurodegeneration. Upregulation of the fusion regulator OPA1 confers a protective effect in cerebellar granular neurons following excitotoxic insult (191), while ablation of DRP1 from forebrain neurons leads to hippocampal atrophy (192). Furthermore, mitochondria are transferred to adjacent neurons following focal cerebral ischemia, and inhibition of transfer worsened neurological outcomes (193). This suggests that the transfer of functional mitochondria from astrocytes to neurons after stroke supports cell viability and recovery. Such a mechanism may even occur in response to the chronic low-grade inflammation in obesity and diabetes in an attempt to protect neurons and therefore warrants further investigation.

## Conclusion and Future Perspectives

While we have some understanding of how mitochondrial dynamics in the brain are associated with food intake and glucose sensing, there are still many more questions to be answered. The cell types involved in the hypothalamus have begun to be dissected, but as yet there is little data on the roles of specific cells in the DVC, and indeed for both regions the role of mitochondria in astrocytes and microglia needs to be further explored. Mitofusins and OPA1 also need to be studied in the DVC in relation to overnutrition, obesity, and T2D. This review has focused on diet-induced obesity and T2D; the genetic etiology of obesity remains unknown, but genome wide association studies (GWAS) of T2D have identified variants associated with T2D (194). Future studies may investigate the role of specific variants and what effects these have on mitochondria in cells of the brain as well as physiological effects on whole body metabolism. Finally, while mitochondrial dynamics are being studied in relation to obesity and T2D, changes to mitochondrial bioenergetics in the brain in these conditions remain unknown and warrant future investigation.

A fine balance of mitochondrial network dynamics is required for healthy brain function, T2D and obesity are modifiable risk factors that if ameliorated could reduce the risk of developing neurodegenerative comorbidities, and targeting mitochondrial dysfunction in the brain could be the link between these disorders. It is clear that mitochondria are crucial in the development and maintenance of obesity and T2D, playing a role in the control of glucose homeostasis and whole-body metabolism across cell types in the hypothalamus and DVC.

## Author Contributions

JH and LN contributed to the initial concepts and wrote the manuscript. BF contributed to the initial concepts and revised the manuscript. All authors contributed to the article and approved the submitted version.

## Funding

Our lab is funded by: Royal Society (RG160605), Diabetes UK (2338) and Wellcome Trust (UNS63234). BF is supported an MRC-Career Development Fellowship (MR/S007288/1).

## Conflict of Interest

The authors declare that the research was conducted in the absence of any commercial or financial relationships that could be construed as a potential conflict of interest.
